# Influence of the activation time of magnesium surfaces on the concentration of active hydroxyl groups and corrosion resistance

**DOI:** 10.1016/j.heliyon.2024.e34772

**Published:** 2024-07-20

**Authors:** Lily Margareth Arrieta Payares, Lizeth Del Carmen Gutierrez Pua, Juan Carlos Rincon Montenegro, Ana Fonseca Reyes, Virginia Nathaly Paredes Mendez

**Affiliations:** a Mechanical Engineering Department, Universidad del Norte, Km5 Vía Puerto Colombia, Barranquilla, 080005, Colombia; b Biomedical Engineering Department, Universidad Simón Bolívar, Barranquilla, Colombia, 080002

**Keywords:** Magnesium alloys, Biodegradable biomaterials, Alkaline treatment, Corrosion resistance, Orthopedic implants

## Abstract

Magnesium alloys have been extensively studied as degradable biomaterials for clinical applications due to their biocompatibility and mechanical properties. However, their poor corrosion resistance can lead to issues such as osteolysis and the release of gaseous hydrogen. This study investigated the influence of the activation time of magnesium surfaces in a sodium hydroxide (NaOH) solution on the concentration of active hydroxyl groups and corrosion resistance. The results indicated that immersion time significantly influences the formation of a corrosion-resistant film and the distribution of surface hydroxyl groups. Specifically, specimens treated for 7.5 h exhibited the highest concentration of hydroxyl groups and the most uniform oxide film distribution. Electrochemical tests demonstrated capacitive behavior and passive surface formation for all evaluated times, with the 7.5-h immersion in NaOH yielding superior corrosion resistance, lower current density, and a more efficient and thicker protective film. SEM and EDS analyses confirmed increased formation of Mg(OH)₂ for samples treated for 5 and 7.5 h, while a 10-h treatment resulted in a brittle, porous layer prone to degradation. Statistical analysis using ANOVA and Fisher's LSD test corroborated these findings. The optimal 7.5-h alkali treatment enhanced magnesium's corrosion resistance and surface properties, making it a promising candidate for orthopedic implants. However, further studies are necessary to assess biocompatibility and physiological responses before clinical implementation.

## Highlights

• NaOH immersion time impacts magnesium alloy's corrosion resistance and surface properties.
• 7.5-h treatment yields the highest hydroxyl group concentration and uniform oxide film.
• Electrochemical tests show capacitive behavior and effective corrosion-resistant film formation.
• SEM and EDS confirm increased Mg(OH)₂ formation after 5 and 7.5-h treatments.
• Statistical analysis validates 7.5-h treatment as optimal for orthopedic implant applications.

## Graphical abstract

**Figure:**
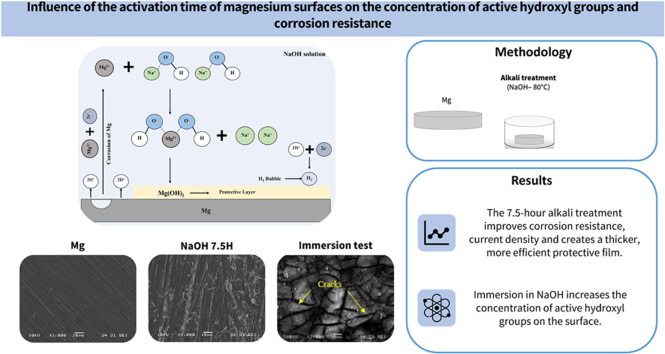

## 1 Introduction

Magnesium and its alloys have been studied as degradable biomaterials for various clinical applications, such as cardiovascular stents, dental cavity fillings, and temporary fixation devices, due to their mechanical properties and excellent biocompatibility. Mg can be absorbed by the human body and even form soluble and non-toxic products that can be eliminated through urine [[1], [2], [3], [4], [5], [6], [7], [8]], Therefore, this material can degrade within physiological environments without posing risks to the patient's health, eliminating the need for a second surgical intervention to remove the implant [8]. However, magnesium alloys have poor corrosion resistance, leading to issues such as osteolysis, mechanical material loss, and rapid and excessive release of gaseous hydrogen, which could result in localized pH increase, delaying the healing process and increasing the risk of bacterial infection around the tissue [[9], [10], [11], [12], [13], [14]].

For this reason, it is necessary to establish strategies that enhance the corrosion behavior of Mg and its alloys. Chemical modification of the surface or deposition of coatings onto magnesium is one of the most studied techniques for orthopedic implants, as it allows for stable and strong bonds to be formed between the surface and the immobilized biomolecule through ionic or covalent chemical bonds capable of withstanding the extreme conditions of the human body during and after implantation [[15], [16], [17], [18], [19]]. However, prior to the deposition of biomolecules, it is essential to prepare the magnesium surface, as direct molecular immobilization or self-assembly is not feasible due to the low bioactivity of the surface, which could lead to a failure in the molecule's self-assembly [20].

The oxide film on metals is typically composed of hydroxyl groups that actively participate in the formation of chemical bonds between the material's surface and biomolecules or silanes, thereby enhancing corrosion resistance and substrate biocompatibility [21]. The number of hydroxyl groups present on the surface is related to the number of charged sites that contribute to interactions with molecules such as proteins, amino acids, and organic acids present in bodily fluids, giving rise to electrostatic forces, ionic interactions, van der Waals bonds, or hydrogen bonding. Therefore, the concentration of hydroxyl groups on the substrate will impact the strength of the interaction, the quantity, and orientation of molecules adhered to the surface, thereby influencing other biological events such as cellular adhesion [22,23].

Previous research has demonstrated that the concentration of hydroxyl groups in the surface oxide film of magnesium (Mg(OH)_2_) can be increased through immersion in a sodium hydroxide (NaOH) solution [[24], [25], [26], [27], [28], [29], [30]]. Alkali treatment not only produces a thin resistive layer that provides greater corrosion resistance to the material but also enhances the surface disposition of magnesium by increasing surface chemical activity and raising surface roughness [20,[31], [32], [33]]. While prior studies have explored various concentrations and durations of NaOH treatment, the innovation of our study lies in assessing the influence of immersion time of a magnesium alloy in a NaOH solution on surface activation. By conducting alkali treatment for durations of 5, 7.5, and 10 h, we aim to comprehensively investigate the optimal conditions for enhancing magnesium alloy performance. Using electrochemical tests and surface characterization, we will evaluate the quantity of hydroxyl groups present on the surface, thus providing valuable insights into optimizing surface modification processes for superior substrate performance.

## 2 Materials and methods

### 2.1 Materials

An Mg alloy (97.76 wt% Mg, 2.24 wt% Al) was used as the substrate for the investigation, and the dimensions of the specimens were defined as required for each characterization technique. All samples were polished with SiC abrasive paper ranging in grain size from 100 to 2000, rinsed, ultrasonically cleaned in 96 % ethanol, and dried with compressed air. For the zinc complex substitution test, the samples were polished to a mirror finish using a 9 μm diamond suspension, followed by a subsequent colloid silica suspension of 0.03 μm [34].

### 2.2 Cleaning and alkali treatment

The specimens were cleaned and activated through alkali treatment. Samples were chemically etched in a solution of NaOH in distilled water at 3 M concentration at 80 °C for three different durations (5, 7.5, and 10 h). Subsequently, the samples were rinsed with 96 % ethanol and dried using compressed air.

### 2.3 Surface characterization and superficial morphology

The chemical composition of Mg was determined using energy-dispersive X-ray spectroscopy (EDS) with a Carl Zeiss EVO MA10 scanning electron microscope and verified through X-ray diffraction (XRD, Bruker, D8 Advance Family) using Cu Kα radiation at 25 mA and 40 kV. The measurement was conducted in a Bragg angle range (2θ) of 10°–80° with a scanning speed of 3°/min. Subsequently, morphological characterization was performed using a scanning electron microscope (SEM, JEOL model 5600).

### 2.4 Chemical characterization

To determine the concentration of active OH groups on the surface, the zinc complex substitution technique described by H. Sakamoto [35] was employed. The samples were immersed in the zinc complex solution for 1 min to create zinc complexes on the Mg(OH)_2_ film. Subsequently, the specimens were washed three times in 150 ml of 96 % ethanol and left in a desiccator for 1 h. Finally, they were immersed in 100 ml of 2.42 mol/L nitric acid for 10 min to release the zinc ions into the solution. The amount of released zinc ions was determined using the Top Wave 06–09 digestion method and quantified by graphite furnace atomic absorption (GFAA). Test samples with a diameter of 16 mm and a height of 8 mm were used for this procedure.

The concentration of active hydroxyl groups, C_OH_ (number/nm^2^), was calculated using the next equation [35]:

Equation 1 $$C_{OH}=\left\{\frac{C_{Zn}\times 10^{-6}\times V\times A}{M\times S}\right\}\times 2$$

where, $C_{Zn}$ is the concentration of zinc ions (ppb), V, the volume of nitric acid (L), S is the surface area of the sample (nm^2^), A is the Avogadro's number ($6.023\times 10^{23}$/mol), and M is the molecular weight of zinc (62.75) [35]. For this test, specimens with a diameter of 16 mm and a height of 8 mm were used.

### 2.5 Immersion test

The initial mass of the control sample and the samples activated for different durations (5 h, 7.5 h, and 10 h) was measured. Subsequently, each sample was immersed in a PBS solution (pH = 7.4) in a hermetically sealed container to prevent evaporation and maintained at (37 ± 1) °C for (7 ± 0.1) days. Daily pH measurements of each immersion solution were conducted. At the end of the 7-day period, the samples were removed from the PBS solution, and the corrosion products were eliminated. Chemical and morphological characterizations were performed using SEM and EDS. The corrosion rate was calculated using equation (2), according to the ASTM standard (ASTM G31), where, w is the weight loss (g), A is the surface area of the specimen (cm^2^), D is the density of the material, 1738 g/cm³, T is the corrosion time (h).

Equation 2 $$\text{Corrosion}\;\text{rate}\;\left(\text{mm}/\text{year}\right)=\frac{\left(8.76\times \hat{10}4\times w\right)}{\left(A\times D\times T\right)}$$

### 2.6 Electrochemical measurements

Electrochemical impedance spectroscopy (EIS) was employed to evaluate the corrosion behavior provided by the alkali treatment, using the GAMRY-Reference 600 Potentiostat/Galvanostat/ZRA system. The specimens were set up in a polymer cell with a three-electrode electrochemical configuration. The treated Mg surface was used as the working electrode (2 cm^2^), Ag/AgCl served as the reference electrode, graphite as the counter electrode, and a 0.9 % NaCl solution was utilized. Prior to measurements, the specimens were immersed in the solution for 10 min at 28 °C to achieve steady-state conditions. Nyquist and Bode diagrams were obtained by performing frequency sweeps in the range of 100,000 kHz to 0.1 Hz, using an alternating current voltage of 10 mV/ms. Corrosion parameters such as corrosion potential ($E_{corr}$) and corrosion current density ($i_{corr}$) were obtained using the Tafel extrapolation method based on Potentiodynamic polarization curves. In this part of the experiment, the specimens were immersed in the NaCl solution for a duration of 20 min at a temperature of 28 °C, aiming to establish steady-state conditions. The corrosion rate according to ASTM G59 using the following equation [36]:

Equation 3 $$CR=3.27\times 10^{-3}\frac{i_{corr}EW}{\rho }$$

Where EW is the equivalent weight of the corroding species in grams and ρ is the density of the corroding material in $g/cm^{3}$. All the tests were repeated three times.

### 2.7 Statistical analysis

The data obtained from the previous tests were evaluated using a one-way analysis of variance (ANOVA) with a confidence level of 95 %. The Fisher's least significant difference (LSD) method was used to compare the means of the evaluated levels (α = 0.05).

## 3 Results and discussion

### 3.1 Surface analysis

**Fig. 1:**
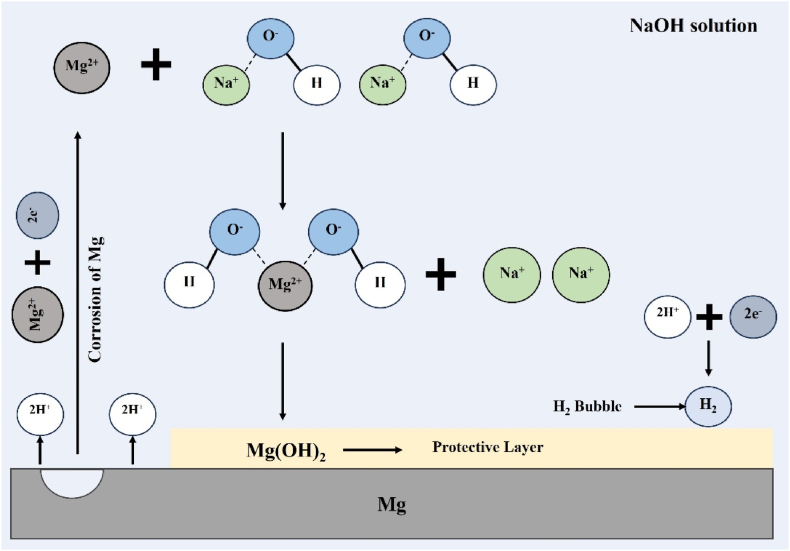
Chemical reaction during alkaline treatment.

The objective of the alkali treatment is to increase the density of available hydroxyl groups on the material's surface. In aqueous solutions, magnesium corrodes rapidly, releasing Mg^+2^ [37,38]. In a NaOH solution, the free Mg ions react with hydroxyl ions (-OH) to form Mg(OH)_2_, which deposits on the material's surface (Equation (5)), creating a protective and porous layer (Fig. 1).

Equation 4 $$Mg\to 2Mg^{+2}+2e^{-}$$

Equation 5 $$Mg^{+2}+2NaOH\to Mg{\left(OH\right)}_{2}+{2Na}^{+}$$

Similarly, during the corrosion reaction, gaseous hydrogen is released as a byproduct in the form of bubbles at the interface of the metal and the solution [37,38].

Equation 6 $${2H}^{+}+2e^{-}\to H_{2}$$

To determine the density of hydroxyl groups (-OH) on the surface, the elemental composition of an area of the surface was characterized for each activation time in NaOH using EDS. Table 1 presents the weight percentage composition of the elements found on the surface of Mg before and after the treatments.

**Table 1:** EDS results.

	Mg (%)	NaOH 5H (%)	NaOH 7.5H (%)	NaOH 10H (%)
**Mg**	98	83.78	68.80	75.25
**O**	2	15.92	31.20	24.75
**Mn**	–	0.30	–	–

After the formation of the magnesium hydroxide layer, a decrease in the elemental Mg percentage is expected as the presence of oxygen on the surface increases. The results show that the oxygen percentage increased with the increase in activation time to 7.5 h and then decreased for the 10-h treatment. Therefore, it can be deduced that there is a higher presence of Mg(OH)_2_ on the surface of the 7.5-h treatment. Furthermore, with a greater presence of the passive layer, a higher number of functional groups will be available on the surface, making it possible to achieve better adhesion of organic molecules in subsequent treatments [39,40].

**Fig. 2:**
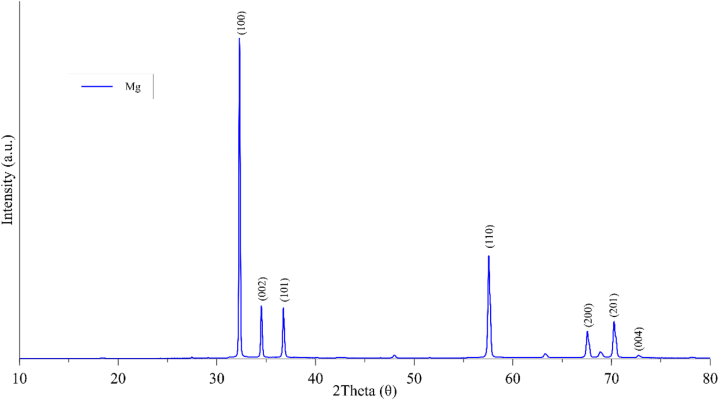
XDR of Mg.

Fig. 2 depicts the X-ray diffraction pattern of magnesium. A hexagonal phase crystalline structure of Mg was obtained, confirmed by the diffraction peak profiles at various lattice planes (100, 002, 101, 110, 200, 201, and 004), corresponding to 2θ values of (32.18, 34.40, 36.62, 57.38, 67.62, 70.02, and 72.51), respectively. These values are consistent with those previously reported in the literature [30].

### 3.2 Zinc complex substitution treatment

To determine the concentration of active hydroxyl groups on the magnesium surface, 50 ml of nitric acid were analyzed after the zinc complex substitution test using atomic absorption spectroscopy. The obtained data are presented in Table 2, From these results, the number of zinc ions in a nm^2^ was calculated, and subsequently, the hydroxyl group data were normalized relative to the concentration obtained in untreated Mg (251.02 N°/nm^2^), as shown in Table 3.

**Table 2:** Concentration of zinc ions in activated Mg.

Sample	Zinc concentration (μg/L)
**Mg**	79.02
**NaOH 5H**	107250
**NaOH 7.5H**	118166.67
**NaOH 10H**	108166.67

**Table 3:** Normalized concentration of active hydroxyl groups.

Sample	$C_{Zn}$	$C_{Zn}$	V	A	S	M	$C_{OH}$	$C_{OH}$
	(μg/l)	(ppb)	(L)	(1/mol)	(nm^2^)	(g/mol)	(Number/nm^2^)	Normalized value
**Mg**	79.02	52.33	0.05	6.02E+2	2.00E+14	62.75	251.02	1
**NaOH 5H**	107250	71026	0.05	6.02E+2	2.00E+14	62.75	340698.42	1357.27
**NaOH 7.5H**	118166.6	78258	0.05	6.02E+2	2.00E+14	62.75	375388.97	1495.47
**NaOH 10H**	108166.6	71636	0.05	6.02E+2	2.00E+14	62.75	343624.48	1368.93

**Fig. 3:**
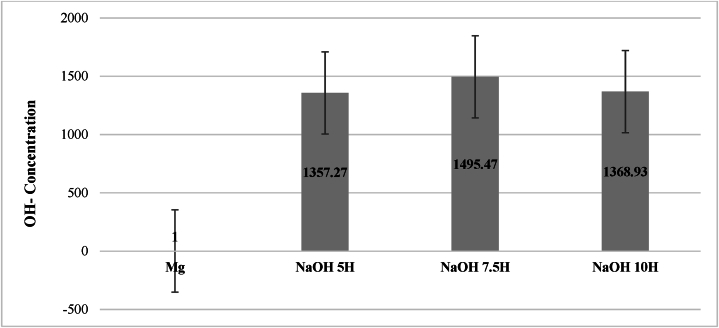
Normalized plot of the concentration of Active Hydroxyl groups.

Fig. 3 displays the normalized values of active hydroxyl group concentration per unit area for the three evaluated times. Following the activation process, a significant increase in hydroxyl groups on the surface is observed, confirming the formation of Mg–OH after alkali treatment for all evaluated times. After 7.5 h of treatment, the highest concentration of –OH groups is evident, with a normalized value of 1495.47. This indicates that a 7.5-h activation results in the greatest number of initiation sites for generating a chemical bond between the Mg surface and silanes or biomolecules. Consequently, this leads to the highest corrosion resistance and biocompatibility of the substrate compared to the other times [21].

### 3.3 Immersion test

**Fig. 4:**
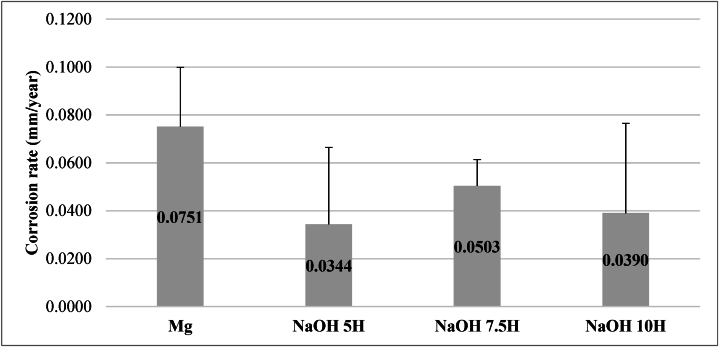
Corrosion rate of Mg without alkaline treatment and sample with alkaline treatment.

The gravimetric results of the specimens subjected to the immersion test did not exhibit statistically significant differences among the specimens activated for different durations (Fig. 4). However, a reduction in corrosion rate is observed for the three treated samples compared to the Mg sample without alkaline treatment. EDS analysis (Table 4) revealed an increase in the concentration of oxygen (O) in the specimens activated for 5 and 7,5 h, suggesting a greater formation of Mg(OH)₂.

**Table 4:** EDS after immersion test.

Sample		EDS	
	O (wt%)	Mg (wt%)	C (wt%)
**Mg**	44.63	42.2	13.18
**NaOH 5H**	56.95	28.74	14.32
**NaOH 7.5H**	55.79	26.86	17.35
**NaOH 10H**	22.47	70.94	6.59

**Fig. 5:**
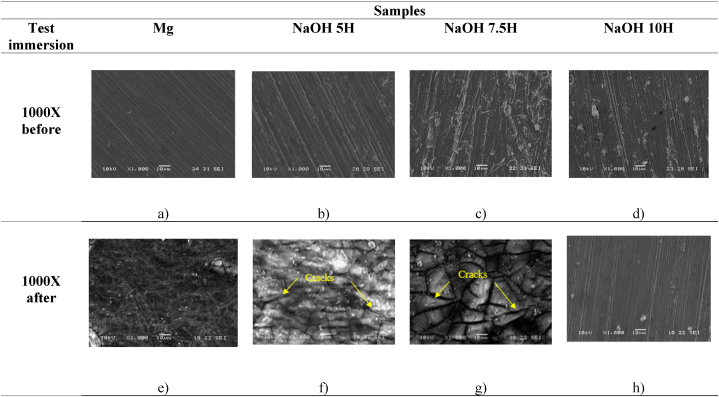
SEM morphologies for the indicated systems before and after the immersion test.

Fig. 5 shows the surface morphology of samples before and after immersion in PBS solution obtained from the scanning electron microscope (SEM). Fig. 5(a–d) reveals the surface topography of the magnesium substrate and the NaOH-treated Mg before the immersion test. All samples exhibit unidirectional lines due to the polishing process. Fig. 5b corresponds to the surface of magnesium treated with NaOH for 5 h, which provided an oxide layer to the surface; however, an uneven distribution of oxide with uncovered areas is evident. Fig. 5d displays samples activated for 7.5 h, showing a uniform oxide layer across the entire surface and thus a homogeneous formation of hydroxyl groups. Finally, Fig. 5d depict samples activated in NaOH for 10 h. The magnifications reveal a greater deposition of oxides in certain areas. Notably, samples activated for 7.5 and 10 h in NaOH resulted in an increased real surface area of the substrate due to surface modification through magnesium oxide [41,42].

Fig. 5(e–h) reveals the surface topography of the magnesium substrate and the NaOH-treated Mg three times after the immersion test. The untreated Mg samples (Fig. 5e) show an uneven oxide distribution. Fig. 5(f and g) corresponding to the magnesium surfaces treated with NaOH for 5 and 7.5 h, reveals a uniform oxide layer across the entire surface. The appearance of cracks is also observed, which may be related to localized corrosion phenomena. This is associated with the oxidation reactions generated during the immersion test, which result in the formation of a layer of Mg(OH)_2_ according to equation (5). Likewise, the brittle morphology observed in Fig. 5(e–g) after the immersion test is due to the properties of fragility and porosity of this layer. As immersion time increases, corrosion gradually spreads, causing the cracks to expand [43,44]. The coating gradually divides into small fragments as the cracks connect, eventually leading to coating failure. Otherwise, after the immersion test, Fig. 5 -f, corresponds to the sample with 10 h of activation A uniform surface rich in Mg is observed, which as reported in Table 3 have also a lower concentration of active OH groups, as reported by the Zn substitution technique.

**Fig. 6:**
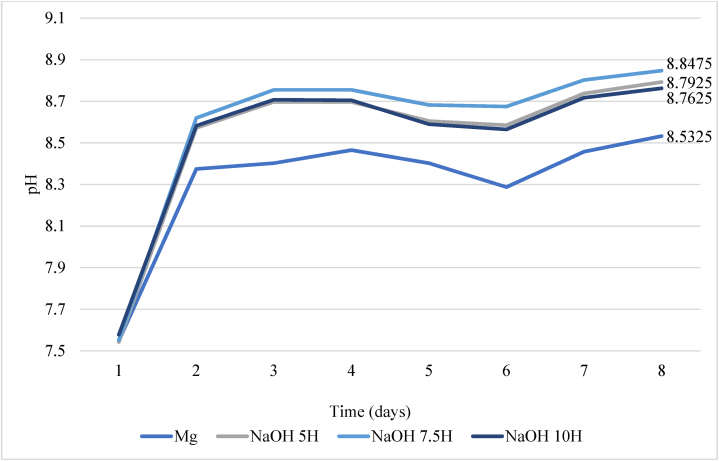
pH values of the PBS during immersion test.

The pH behavior over the exposure time at the surface-electrolyte interface was consistent with previously reported studies, Fig. 6 shows that pH increased from 7,5 to approximately 8,8 after 24 h of immersion. This increase can be attributed to the formation and dissolution of the Mg(OH)₂ film, and consequently, the release of OH⁻ into the electrolyte, reaching the maximum potential corresponding to the stabilization of hydroxylated species [45]. Given that the samples were activated in an aqueous NaOH solution and the immersion test was conducted in a neutral solution, the results can be explained by the presence and subsequent release of OH⁻ groups from the Mg(OH)₂ layer when the samples are immersed in the PBS solution (pH 7.4). As previously mentioned in the chemical equation of the transformations occurring during activation, a porous and fragile Mg(OH)₂ film forms, allowing the easy release of OH⁻ when the samples are exposed to a neutral pH due to the high reactivity of these hydroxyl groups. This additionally contributes to the increase in pH, catalyzing the corrosion reaction [46,47].

Moreover, the porosity of the layer favors the dissolution of Mg and Mg^2^⁺, as it allows the immersion fluid to react with the underlying Mg, similar to what was observed in the non-activated samples. This dual effect – the release of OH⁻ from the Mg(OH)₂ layer and the reaction of the underlying Mg – resulted in a faster reaction during the immersion test. This increase in reaction rate is consistent with the activation objective, as these findings indicate that the samples activated for this duration have a greater propensity to interact with functional agents in biomedical applications. Additionally, it highlights that since the activation process can be the initial phase of functionalization processes, subsequent stages should aim to form uniform coatings to reduce the likelihood of the Mg(OH)₂ layer dissolution.

### 3.4 Electrochemical measurements

**Fig. 7:**
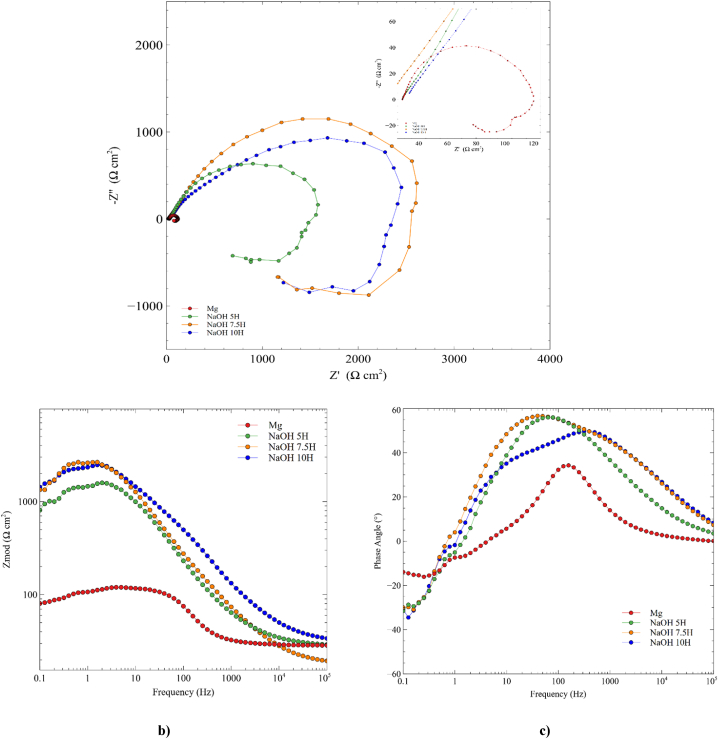
Electrochemical measurements: a) Nyquist diagram, b) Bode plots of Log∣z∣ and c) bode plots of phase angle of untreated and treated Mg.

Fig. 7 depicts the Nyquist and Bode diagrams of the evaluated systems. The Nyquist plots for all activation times exhibit a capacitive loop with a diameter larger than that of the Mg substrate, demonstrating the presence and efficiency of the coating through reduced charge transfer processes (Fig. 7a). Additionally, an inductive loop is evident, which may be associated with pitting corrosion, the adsorption or desorption of intermediate products like magnesium oxides, magnesium hydroxides, or hydrogen ions generated on the electrode surface due to electrochemical reactions occurring during corrosion or accelerated anodic dissolution [48,49]. The assessment of different activation times revealed that the 7.5-h activation time offered the largest diameter in the capacitive loop, indicating better corrosion behavior compared to the other tested systems.

The Bode diagram illustrates the variation of impedance modulus with frequency for the Mg/NaOH-Electrolyte interface. In Fig. 7b, the impedance modulus is higher for all activation times compared to the Mg substrate at low frequencies, once again confirming the presence of the passive layer and the delay of corrosion reactions [49,50]. At low frequencies, untreated Mg exhibited a higher phase angle; however, at high frequencies, the 7.5 and 10-h activation times showed the highest phase angle (Fig. 7c). A larger phase angle implies more capacitive behavior and a more passive surface, leading to slower charge transfer processes and greater corrosion resistance of magnesium [[49], [50], [51], [52]]. Among the evaluated times, the 7.5 and 10-h treatments exhibited the highest phase angle. Considering that both phase angle and impedance modulus depend not only on the thickness but also on the uniformity of the coating, it can be inferred that these two activation times offer a uniform and passive layer [53]. The results obtained from the Nyquist and Bode diagrams confirm the effectiveness of passivation on Mg against corrosion reactions.

**Fig. 8:**
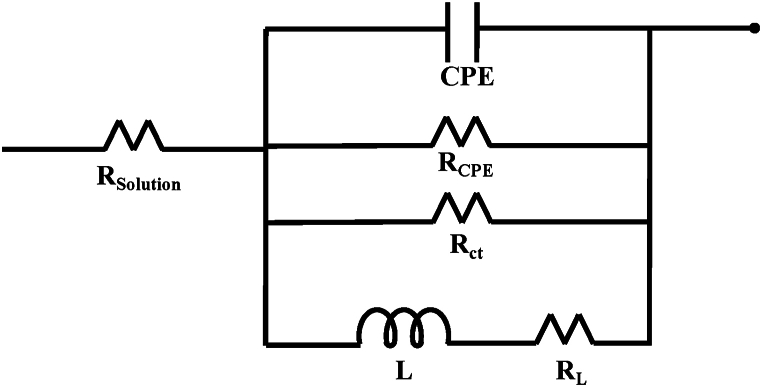
Equivalent circuit for fitting the results.

Fig. 8 illustrates the equivalent electrical circuit employed for fitting the experimental electrochemical impedance data of Mg with and without treatment. Table 5 presents the fitting parameters, where R_solution_ represents the resistance provided by the electrolyte, CPE and R_CPE_ correspond to a constant-phase element and electrochemical double-layer resistance, respectively. R_ct_ represents the charge transfer resistance, L is inductance, and R_L_ is inductive resistance [54].

**Table 5:** Electrochemical impedance parameters of treated and untreated Mg.

Samples	R_Solution_	R_CPE_	CPE	R_ct_	R_L_	L	R_p_
	(ohm)	(ohm)	(μF/cm^2^)	(ohm)	(ohm)	(H)	(ohm)
**Mg**	30	100	20.7	883.8	70	100	64.86
**NaOH 5H**	30.31	14790	35.4	1.93 E+03	803.8	1129	567.03
**NaOH 7.5H**	20.06	7619	16.9	5.44 E+03	2311	2108	1621.70
**NaOH 10H**	28.73	16430	30.24	6.51 E+03	1812	2829	1417.46

Using these parameters, the polarization resistance (R_p_) associated with surface modification due to alkali treatment was calculated to study the corrosion behavior at the different evaluated treatment times, as shown below [48].

Equation 7 $$R_{P}=\frac{R_{ct}*R_{L}}{R_{ct}+R_{L}}$$

The results indicate that the highest Rp value of approximately 1621.70 Ω was achieved for the 7.5-h treatment time, followed by the 10-h treatment with 1417.46 Ω, and the lowest Rp was reported for the 5-h treatment time. In this regard, it can be concluded that alkali treatment for 7.5 h induces a more efficient chemical modification against corrosion processes, thus providing greater protection to magnesium.

#### 3.4.1 Polarization curves

**Fig. 9:**
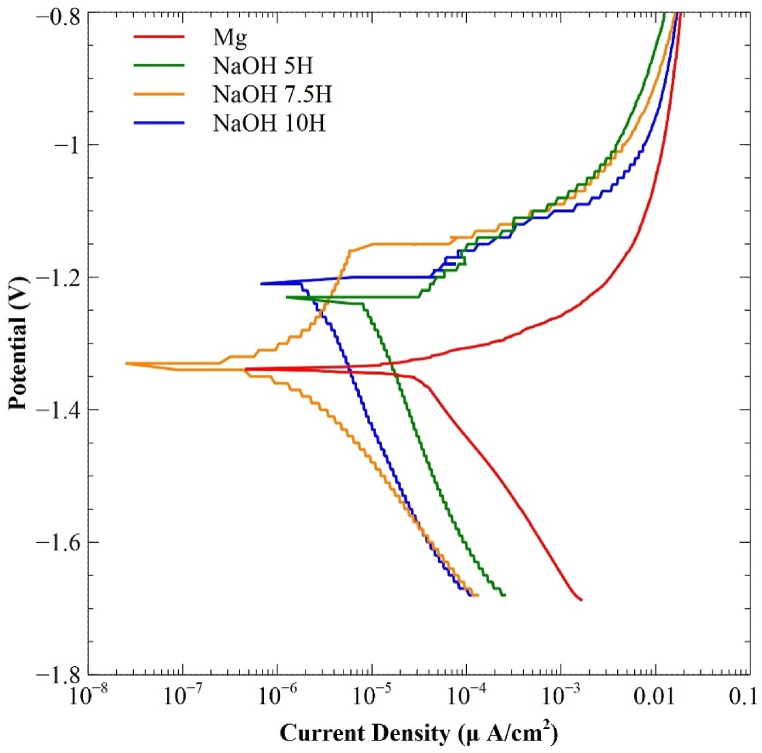
Potentiodynamic polarization curves of Mg with and without alkali.

Fig. 9 displays the polarization curves for the different activation times, along with the untreated Mg curve. Table 6 presents the parameters related to the corrosion kinetics of the evaluated systems. The results show a shift of E_corr_ towards nobler values for the 5 and 10-h treatments, while the 7.5-h treatment exhibited a slight shift towards more negative values compared to the untreated substrate. The corrosion rate for all evaluated treatment times was lower than that of untreated Mg. However, the 7.5-h time displayed the lowest current density (1.04 μA/cm^2^) and consequently the lowest corrosion rate of 0.089 mm/year, which is 96 % lower than the corrosion rate of untreated Mg.

**Table 6:** Electrochemical measurements derived from the polarization curves of treated and untreated Mg.

Sample	Mg	NaOH 5H	NaOH 7.5H	NaOH 10H
**β**_**A**_**(V/decade)**	7.18E-02	7.30E-02	3.37E-01	8.92E-02
**β**_**C**_**(V/decade)**	2.19E-01	3.74E-01	2.04E-01	3.52E-01
**I**_**corr**_**(μA/cm**^**2**^**)**	17.8	4.86	1.04	1.425
**E**_**corr**_**(V/Ag–AgCl)**	−1.29	−1.24	−1.33	−1.21
**Corrosion Rate (mm/year)**	2.355	0.534	0.0894	0.134

#### 3.4.2 Efficiency, capacitance, and thickness of the protective film

The efficiency and thickness (metal-oxide-electrolyte interface of the electrochemical systems) of the generated coatings were evaluated using equations Equation (8) and Equation (9) respectively.

Equation 8 $$\eta =\frac{R_{P-activación}-\;R_{P-Mg\_base}}{R_{P-Mg\_base}}\;\%$$

Equation 9 $$d=\varepsilon \varepsilon_{0}S/Q$$

Where $R_{p}\text{,}$ Q, $R_{ct}$ represent the previously explained parameters in the equivalent circuit, and ε is the relative dielectric constant of magnesium oxide (ε = 10). $\varepsilon_{0}$ is the vacuum permittivity ($\varepsilon_{0}=8.85\times 10^{-14}$ F/cm) [48,55] and S is the exposed area.

The sample activated for 7.5 h exhibits the best characteristics of the film, showing a 2400 % increase in polarization resistance and a coating thickness of 52.4 nm (Table 7). These results align with the data obtained from the EIS test.

**Table 7:** Characteristics of the activated Mg film.

Sample	*R*_*p*_	*η*	*Q*	*d*
	(ohm)	%	(μF/cm^2^)	nm
**Mg**	64.86		20.7	
**NaOH 5H**	567.03	774 %	35.4	25
**NaOH 7.5H**	1621.7	2400 %	16.9	52.4
**NaOH 10H**	1417.46	2085 %	30.24	29.3

### 3.5 Statistical analysis

A one-factor analysis of variance (ANOVA) was conducted to determine if the duration of alkali treatment time influences the corrosion resistance provided by the coating. The results indicated that, with a 95 % confidence level, there is a difference in the resistivity of the Mg–OH film induced by the material's activation time (P-value: 1.44E-07). Next, the Fisher's Least Significant Difference (LSD) method was employed to assess whether there are differences in resistance based on the treatment time and to identify resistivity levels in terms of immersion times. The results are presented in Table 8. Three resistivity levels were identified. When comparing the mean pairs of resistances obtained for the three activation times, a homogenous zone of high resistivity was identified for alkali treatments at 7.5 and 10 h. Therefore, the corrosion resistance obtained from these two methods is statistically equal and superior to the resistivity provided by a treatment time of 5 h and untreated Mg.

**Table 8:**
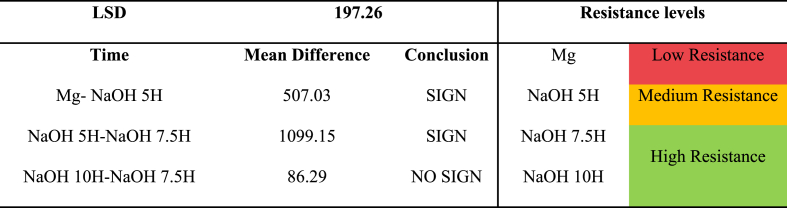
LSD (Least Significant Difference) of activation time.

## 4 Conclusion

Based on the results obtained in this study, it can be concluded that the immersion time of a magnesium alloy in an alkali treatment using a NaOH solution significantly influences the material's corrosion behavior and surface disposition for subsequent treatments. For all evaluated times, the presence of a magnesium oxide layer on the surface and an increase in the concentration of active hydroxyl groups compared to untreated magnesium were observed. Particularly, specimens treated for 7.5 h exhibited the highest concentration of hydroxyl groups and an improved distribution of the oxide film on the surface.

Electrochemical tests demonstrated that all evaluated times result in a capacitive behavior and a passive surface, indicating the formation of an effective corrosion-resistant film compared to untreated magnesium substrate. Likewise, Nyquist plots and polarization curves showed that the 7.5-h immersion time yielded the best results in terms of corrosion resistance, current density, and a more efficient and thicker protective film.

The evaluation of the immersion test demonstrated that the activation of magnesium alloys treated with NaOH for 7.5 h provided a more uniform and stable Mg(OH)₂ layer. The pH monitoring revealed a significant increase due to the release of OH⁻ groups. SEM and EDS analyses corroborated the greater formation of Mg(OH)₂ in the samples activated for 5 and 7.5 h. The specimens treated for 10 h exhibited a brittle and porous layer, susceptible to accelerated degradation, suggesting that an optimal activation time is crucial for improving the durability and functionality of magnesium alloys in biomedical applications.

ANOVA confirmed the influence of immersion time on magnesium's corrosion behavior, and the LSD analysis identified a region of high resistivity for 7.5 and 10-h times.

In conclusion, alkali treatment for 7.5 h in a NaOH solution proved to be the most effective in enhancing magnesium's corrosion resistance through the formation of a more uniform oxide film and improving surface disposition for subsequent treatments, such as biomolecule adhesion. These findings are relevant to the study of using this alloy in orthopedic implants due to the enhancements in material surface properties. However, further studies are required to evaluate biocompatibility and responses under physiological conditions before considering clinical implementation.

## Funding

The authors acknowledge the financing of the:

• Ministry of Science, Technology, and Innovation of Colombia through the “Créditos educativos condonables para la formación de capital humano de alto nivel para las regiones” - Atlántico, Colombia. National master's modality. CALL No. 809 of 2018.
• Biotechnology laboratory of Universidad del Norte.

## Data availability

The data that support the findings of this study are available from the corresponding author upon reasonable request. As the data are part of an ongoing research project, access to the data will be provided in a manner that ensures the integrity and confidentiality of the research. Further information and requests for resources and reagents should be directed to and will be fulfilled by the lead contact, Lizeth Gutierrez Pua (Lizeth.GutierrezPua@UTDallas.edu, gutierrezdl@uninorte.edu.co).

## Ethical approval

No animal or human tissue experiments were carried out in this research. No human subjects were included in this study.

## CRediT authorship contribution statement

**Lily Margareth Arrieta Payares:** Writing – original draft, Visualization, Methodology, Investigation, Formal analysis, Conceptualization. **Lizeth Del Carmen Gutierrez Pua:** Writing – review & editing, Writing – original draft, Visualization, Methodology, Investigation, Formal analysis, Conceptualization. **Juan Carlos Rincon Montenegro:** Writing – review & editing, Writing – original draft, Visualization, Methodology, Investigation, Formal analysis, Conceptualization. **Ana Fonseca Reyes:** Writing – review & editing, Formal analysis. **Virginia Nathaly Paredes Mendez:** Writing – review & editing, Supervision, Formal analysis, Conceptualization.

## Declaration of competing interest

The authors declare the following financial interests/personal relationships which may be considered as potential competing interests:Lily Margareth Arrieta Payares reports financial support was provided by Ministry of Science, Technology, and Innovation of Colombia.
